# Intussusception among Children Admitted in the Department of Pediatric Surgery of a Tertiary Care Centre: A Descriptive Cross-Sectional Study

**DOI:** 10.31729/jnma.7994

**Published:** 2023-02-28

**Authors:** Ramana Rajkarnikar, Suraj Singh, Mridul Prasad Joshi, Anuj Kayastha

**Affiliations:** 1 Department of Pediatric Surgery, International Friendship Children's Hospital, Kathmandu, Nepal; 2 Department of Pediatric Surgery, Kanti Children's Hospital, Kathmandu, Nepal

**Keywords:** *intussusception*, *laparotomy*, *paediatrics*, *prevalence*, *ultrasound*

## Abstract

**Introduction:**

Intussusception is the second most common cause of acute abdominal pain in infants and preschool children. The aetiology for intussusception is idiopathic at this age. Hydrostatic reduction and exploratory laparotomy with proceed are the options for the management of intussusception. The aim of this study was to find out the prevalence of intussusception among patients admitted to the Department of Pediatric Surgery of a tertiary care centre.

**Methods:**

This is a descriptive cross-sectional study conducted among admitted patients in the Department of Pediatric Surgery of a tertiary care centre after ethical approval from the Ethical Committee (Reference number: A37-77/78). Data were collected from 1 January 2018 to 31 December 2020 from admitted children aged 6 months to 5 years of age. Data collection was done from the hospital record section using the convenience sampling method. Point estimate and 95% Confidence Interval were calculated.

**Results:**

Among 1785 admitted patients, 267 (14.96%) (13.31-16.61, 95% Confidence Interval) were found to have intussusception. Among them, the hydrostatic reduction was successful in 246 (92.13%). Meanwhile, 21 (7.86%) of cases underwent laparotomy. The peak age of patients was seen in 148 (55.43%) in the age group of 1-3 years.

**Conclusions:**

Intussusception is one of the common surgical emergencies in children. Hydrostatic reduction of intussusception is a simple and effective method for the treatment of intussusception in children.

## INTRODUCTION

Intussusception is defined as an invagination or telescoping of the proximal segment of the bowel into an adjacent distal portion of the bowel leading to obstruction, strangulation, ischemia and eventually necrosis of the bowel.^1-3^ In children, it is the second most common cause of acute abdominal pain.^1^

Treatment options for intussusception are both nonoperative and operative. In 1982, Kim and his group did the first Ultrasound guided hydrostatic reduction with normal saline.^4^ The procedure may be performed with water, saline or Hartmann solution.^2^

The aim of this study was to find out the prevalence of intussusception among patients admitted to the Department of Pediatric Surgery of a tertiary care centre.

## METHODS

This was a descriptive cross-sectional study done in the Department of Pediatric Surgery of International Friendship Children's Hospital (IFCH), Kathmandu, Nepal. Ethical approval was obtained from the Ethical Committee of the hospital (Reference number: A37-77/78). All the patients admitted to the Department of Pediatrics from 1 January 2018 to 31 December 2020 with ages ranging from 6 months to 5 years were included in the study. Cases with signs of peritonitis marked abdominal distension and radiological evidence of free peritoneal air have been excluded. A convenience sampling method was used. The sample size was calculated using the formula:


$$\begin{array}{ll} \text{n} & ={\text{Z}}^{2}\times \frac{\text{p}\times \text{q}}{{\text{e}}^{\text{2}}}\\ & =1.96^{2}\times \frac{0.5\times 0.5}{0.03^{2}}\\ & =1068 \end{array}$$

Where,

n = minimum required sample size
Z = 1.96 at 95% of Confidence Interval (CI)
p = prevalence taken as 50% for maximum sample size calculation
q = 1-p
e = margin of error, 3%

The calculated minimum required sample size was 1068. After adding 20% for missing data, the adjusted sample was 1334. However, we have included 1785 patients in the study.

Intussusception was diagnosed clinically and on an ultrasound scan. Data regarding age, sex, and presenting symptoms with the duration of onset of symptoms were analysed. The Criteria for successful reduction were the disappearance of the intussusception through the ileocecal valve and a clear demonstration of the opening of the ileocecal valve to allow passage of fluid and air bubbles from the cecum to the terminal ileum. A maximum of 3 attempts of reduction were tried. If there was the persistence of intussusception and were no signs of reduction as mentioned above, failure of reduction was confirmed.^1,5^ The data were entered and analysed in IBM SPSS Statistics version 20.0. Point estimate and 95% CI were calculated.

## RESULTS

**Figure 1:**
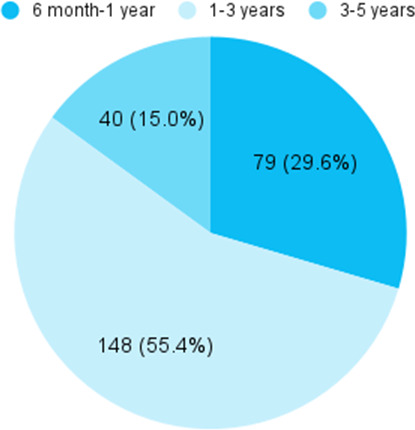
Distribution of patients according to age (n= 267).

Among 319 gynaecological patients, hysteroscopy was done Out of 1785 patients, 267 (14.96%) (13.31-16.61, 95% CI) were found to have intussusception. The total number of cases undergoing hydrostatic reduction in 2018, 2019 and 2020 is 97 (36.33%), 81 (30.33%) and 89 (33.33%) respectively. A total of 61.05 % (163) were male and 38.95 % (104) were female with male to female ratio of 1.56:1. Patients of age group 6 months to 1 year were 79 (29.59%), from 1-3 years were 148 (55.43%) were from the 1-3 years age group and 40 (14.98%) were from 3-5 years age group (Figure 1).

**Figure 2:**
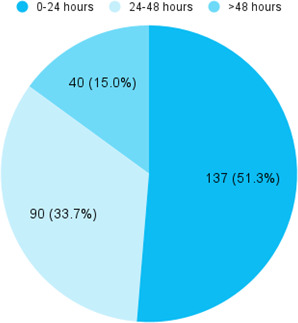
Duration of presentation to the hospital with clinical features (n= 267).

The number of patients with features suggestive of intussusception presented to the emergency department from the time of onset of symptoms within 0-24 hours, 24-48 hours and more than 48 hours are 137 (51.31%), 90 (33.7%), 40 (14.98%) (Figure 2).

Among the presenting complaints, the intermittent cry was the most common complaint reported in 193 cases (72.28%), followed by abdominal pain in 180 cases (67.41%), vomiting in 170 cases (63.67%) and per rectal bleeding in 60 cases (22.47%) (Table 1). The classical triad of intussusception i.e. abdominal pain, vomiting and per-rectal bleeding was present in 9.64 % of cases.

**Table 1:** Variation of the clinical features in patients of Intussusception (n= 267).

Clinical features	n (%)
Intermittent cry	193 (72.28)
Abdominal pain	180 (67.41)
Vomiting	170 (63.67)
Per-rectal bleeding	60 (22.47)

Hydrostatic reduction was successful in 246 (92.13%) patients. A total of 19 patients (7.72%) who had successful reduction had a recurrence that was treated with a repeat hydrostatic reduction. No bowel perforation occurred in repeat reduction. Patients with failed reduction at 3 attempts were managed surgically. All of these patients underwent exploratory laparotomy and 7 (33.33%) patients required manual reduction only while 14 (66.67%) patients required bowel resection and anastomosis.

## DISCUSSION

Intussusception is a common emergency in infants and children.^4^ The prevalence of intussusception in our study was 14.96% among admitted children and there is no such study done which describe the absolute prevalence of intussusception in hospital setting. However in a study incidence rate of intussusception was 21 per 100,000 children aged ≤15 years.^6^ Also, in another study the incidence of intussusception was found to be 31.61 per 100,000 in children less than 5 years old.^7^ It is more common in boys than girls. In our study, the ratio of male to female was 1.56:1 which is almost equal to a published study (i.e. 1.78:1).^8^ The most common age group diagnosed with intussusception in our study is 1-3 years while in an another study, the common age group affected was 6-11 months.^9^

The symptoms of intussusception are non-specific and the child may present with a variety of symptoms.^3,4^ In our study, intermittent cry remains the most common presenting feature while in some studies have shown that paroxysmal abdominal pain was the most common presenting feature.10 This could be because of the age-wise variation in the number of patients. In our study, 9.64% of the children presented with classical triad colicky abdominal pain, bloody stool and vomiting which is similar to published literature. (i.e. 9.3 %).^10^

Though intussusception was first described by Paul Barbette in 1692, it took over 3 centuries before its sonographic features were described in 1977. Raffainsperger has written that as far back as Hippocrates' time, there were allusions to a reduction of intussusception by the injection of air/fluid into the rectum. In 1836, Samuel Mitchell reported non-surgical reduction of intussusception. In 1876, Hirschsprung published the first of a series of reports on hydrostatic reduction with results of 23% mortality that was superior to operative treatment until the mid-1900s.^1^ Hipsley (1926) published a series of 100 cases, 62 of which were treated by hydrostatic saline enema, with only 1 death.^11^

The use of US-guidance hydrostatic reduction permits an even more liberal approach to enema therapy owing to the lack of radiation exposure in comparison to reduction under fluoroscopy.^2,12,13^ US-guided hydrostatic reduction is a relatively simple and safe procedure. It is associated with less morbidity and patients have a shorter hospital stay. Thus, lead to cost savings for the health care system.^9^ Hydrostatic reduction in our study was effective in 92.1% cases while in some study hydrostatic reduction was effective in only 65% cases.^5^ Also, in study done in Nepal, the pneumatic reduction was attempted and it was successful in 92% cases which is similar to the success rate of hydostatic reduction like in our study.^14^

The goal of any type of enema therapy is to reduce the intussusception by exerting pressure on the apex of the intussusceptum to push it from pathological position into the original position.^2^ It has been found empirically that intracolonic pressure reaches a plateau during hydrostatic enema therapy. This pressure is more constant than that exerted during air insufflation in which there tend to have fluctuation in the intraluminal pressure that can surpass the pressure threshold. Such pressure fluctuation increase the risk of perforation in pneumatic reduction.^15^ Hence advantages of using air for treatment of intussusception have been questioned recently, with the most serious charge being a greater risk of perforation and the possibility of developing a tension pneumoperitoneum.^4^ In our study there has been no case of perforation. Also, use of sedation had been thought to improve the reduction rate.^15^

Surgery is now the accepted backup after radiologic-guided reduction of intussusception.^1^ It is usually reserved for children presenting with long-duration of symptoms and failed hydrostatic/pneumatic reduction.^12^ In our study 21 cases had failed reduction and had to undergo surgery. Of which 7 cases had successful open reduction and 14 cases needed resection and anastomosis.

Since this study was conducted in one centre, it cannot reflect the generalized scenario of the effectiveness of hydrostatic reduction in the other healthcare setting of the country and also the data are regenerated by reviewing the record book. Hence, the result lacks external validity.

## CONCLUSIONS

Intussusception is one of the common surgical emergencies in children. Non-operative management i.e. US-guided hydrostatic reduction with normal saline is a simple and highly effective method for the treatment of intussusception and the recurrence of intussusception remains lower with the hydrostatic eduction. There are no such studies that reflect the prevalence of intussusception in a hospital setting. So, more studies need to be done to find out the absolute prevalence of intussusception.
